# Optimizing an efficient ensemble approach for high-quality de novo transcriptome assembly of *Thymus daenensis*

**DOI:** 10.1038/s41598-023-39620-6

**Published:** 2023-07-31

**Authors:** Hosein Ahmadi, Morteza Sheikh-Assadi, Reza Fatahi, Zabihollah Zamani, Majid Shokrpour

**Affiliations:** Department of Horticulture Science, Faculty of Agriculture and Natural Sciences, University of Tehran, Karaj, Iran

**Keywords:** Biotechnology, Computational biology and bioinformatics, Plant sciences

## Abstract

Non-erroneous and well-optimized transcriptome assembly is a crucial prerequisite for authentic downstream analyses. Each de novo assembler has its own algorithm-dependent pros and cons to handle the assembly issues and should be specifically tested for each dataset. Here, we examined efficiency of seven state-of-art assemblers on ~ 30 Gb data obtained from mRNA-sequencing of *Thymus daenensis*. In an ensemble workflow, combining the outputs of different assemblers associated with an additional redundancy-reducing step could generate an optimized outcome in terms of completeness, annotatability, and ORF richness. Based on the normalized scores of 16 benchmarking metrics, EvidentialGene, BinPacker, Trinity, rnaSPAdes, CAP3, IDBA-trans, and Velvet-Oases performed better, respectively. EvidentialGene, as the best assembler, totally produced 316,786 transcripts, of which 235,730 (74%) were predicted to have a unique protein hit (on uniref100), and also half of its transcripts contained an ORF. The total number of unique BLAST hits for EvidentialGene was approximately three times greater than that of the worst assembler (Velvet-Oases). EvidentialGene could even capture 17% and 7% more average BLAST hits than BinPacker and Trinity. Although BinPacker and CAP3 produced longer transcripts, the EvidentialGene showed a higher collinearity between transcript size and ORF length. Compared with the other programs, EvidentialGene yielded a higher number of optimal transcript sets, further full-length transcripts, and lower possible misassemblies. Our finding corroborates that in non-model species, relying on a single assembler may not give an entirely satisfactory result. Therefore, this study proposes an ensemble approach of accompanying EvidentialGene pipelines to acquire a superior assembly for *T. daenensis*.

## Introduction

*Thymus* (Lamiaceae) is a genus of more than 250 species in the world^1^. *Thymus daenensis* Celak., an indigenous thyme species, now has a dramatically dangerous status^1^. This species is found primarily at high elevations in the Zagros Mountains^2^. Unfortunately, the plant survival has been compromised by annual over-exploitation of natural habitats, excessive grazing, and natural disasters such as forest fires, floods, and droughts. Indeed, such harsh conditions severely threaten its limited populations^2^. *T. daenensis* is a medicinally active herb well-known as a rich source of monoterpene phenolics (thymol and carvacrol)^3^. The plant is widely used in the pharmaceutical industry due to its expectorant and anti-inflammatory properties. It is also a popular herb in kitchens with ample use as a culinary and flavoring agent^3^.

In competition with commonly cultivated thyme species (i.e., *T. vulgaris*), *T. daenensis* possesses some superior characteristics, such as higher amount of essential oil, higher thymol content, and better stress tolerance^4^. These characteristics demonstrate the great potential of the plant to form the nuclei of primary selections in order to acquire a drug yield greater than that of *T. vulgaris* cultivars^4^. However, due to the absence of genomic resources and a lack of functional genomic studies, progress in the molecular breeding of this species has been delayed^3^.

Recent advances in the development of innovative sequencing platforms and the emergence of revolutionizing technologies with reduced costs and labor have paved a bright route for generating massive amounts of genetic data^5^. Since genome drafting is a time-consuming process, mRNA sequencing can support a helpful and time-effective procedure to capture a rapid snapshot of real-time transcript pools for various samples under any given condition^6,7^. With the advent of the post-genomic era, transcriptome sequencing has enabled researchers to discover alternative isoforms, reconstruct full-length transcripts, and quantify spatiotemporal catalogs of mRNAs^8^. Mining transcriptional libraries in the absence of a reference genome can be definitely an applicable tool for exploring the overall genome information of an organism^9^.

De novo assembly of transcriptomes is highly recommended, even when a reference genome is present^9^. Because it is an influential leverage to recover new alternative variants, unique nucleotide polymorphs, and genes hidden behind missing regions of a preliminary genome^9^. To these ends, transcriptome profiling of medicinal plants has aggregated twin foci of consideration because of its promising aid in monitoring biosynthesis pathways of secondary metabolites and genes involved in controlling plant growth^10^. On the other hand, its valuable contribution to the development of molecular markers based on simple sequence repeats (SSRs) helps remarkably to accelerate ongoing breeding programs^11^.

Nonetheless, the initial assembly phase required for future studies is still encountered with some unique challenges^12^. Indeed, the generation of an optimized, precise, and high-quality assembly of RNA-seq reads is an imperative prerequisite for unbiased analysis of downstream pathways^10,11^. Varying expression levels, highly similar isoforms stemming from gene paralogues, and shared exon regions among alternative variants are all sets of circumstances that make the whole procedure complicated^12–14^. For instance, different polyploidy levels, gene duplication events, and the presence of various kinds of transcript isoforms for a singular gene are some sorts of difficulties in assembly procedure of thyme species transcriptome^15,16^.

De novo transcriptome assemblers appealing to *de Bruijn* graphs (e.g., Trinity^17^, Velvet-oases^18^, IDBA-Trans^19^, and rnaSPAdes^20^) are inevitably dealing with sequencing errors (which potentially generate false k-mers or graph nodes), repetitive segments, and bioinformatic artifacts^21^. These errors can significantly distort the valid trajectory of any transcriptome assembly and ultimately result in erroneous modeling of complex genes^22–25^. Therefore, no single ideal assembler is recommended by bioinformaticians for all ranges of input dataset^26^. Users often try to proceed with the research by relying solely on a single assembler without taking into account that each tool has its computational strengths and weaknesses^26^. For instance, IDBA-trans, as a sensitive assembler to low expressed genes, has been developed based on a probabilistic progressive approach to iteratively eliminate the incorrect edges^19^. This software retains the correct vertices of low-expression isoforms^19^. Velvet-Oases can resolve repetitive regions and account for the uneven expression of alternative isoforms but produces more chimeric structures^18^. BinPacker^27^ was also introduced to improve possible weaknesses in Trinity^17^ and Bridger^28^. This package is designed to integrate coverage information and solve a series of bin packaging problems in order to correctly retrieve a set of paths on splicing graphs^27^. The goal of the Binpacking algorithm is to retrieve a valid set of transcripts through overlapping or junction areas by optimizing the contour-path-cover of each splicing graph^27^.

To benefit from each assembler, one common method is to combine (concatenate) the outputs of multiple assemblers^29,30^. However, this approach may result in lengthy, poorly assembled transcripts^31^. In this respect, EvidentialGene was designed to target assembly-related issues^32^. This package intends to eliminate redundancy and exclude misassembled transcripts by focusing on coding regions (CDS)^32^. CAP3, another useful tool used in the ensemble approach, has been mainly applied to cluster and fuse similar isoforms into a consensual sequence^29^.

Any critical investigator can keep this question in mind "what method of assembly is instructive to my study?". The root can be traced back to the lack of a comprehensive method for benchmarking and quality assessment of assembly datasets^33^. Numerous reference-based and reference-free metrics have already been evolved to control the quality of a transcriptome assembly^29^. Users typically evaluate the outputs of these programs by estimating the number of complete transcripts, Nx statistics, mapping rates, and benchmarking of single-copy orthologs (BUSCO)^33^. The algorithms employed by each bioinformatic tool have their unique efficacy^26^. Generally, there is no indisputable agreement on "which metric has a precedent to serve as a good referee?". Therefore, it would make sense to apply multiple criteria for making a decision and assigning a concise judgment^26^.

This study generated 30 Gb high-quality transcriptomic data for *Thymus daenensis* Celak, a valuable medicinal species without a reference genome. We developed an ensemble workflow to attain a non-redundant and well-optimized transcriptome assembly for this species. In this workflow, we integrated five single assembler programs and two combining tools (CAP3 and EvidentialGene) following the concatenation approach, each with its own unique algorithmic design. The main algorithm behind the basis of developed assemblers (such as Trinity, IDBA-Trans, rnaSPAdes, BinPacker, Velvet-Oases) is *de Bruijn* graphs algorithm, but each has its own focus and variations on greedy k-mer extension, overlap-layout-consensus, exhaustive enumeration and other statistical strategies. Additionally, CAP3 follows the overlap-layout-consensus (OLC) algorithm, and EvidentialGene employs sequence alignment and homology search^17–20,27,29,32^. The current work addresses the following questions: (1) What parameters should be considered in a standard quality control pipeline? (2) Which assembly workflow is an appropriate method; single-assembly or ensemble approach? (3) Which software is the winner of the assessments through normalized indices? (4) And, which one yields higher blast hits and lower misassembled transcripts?

## Results

### Transcriptome construction quality and statistics

**Figure 1:**
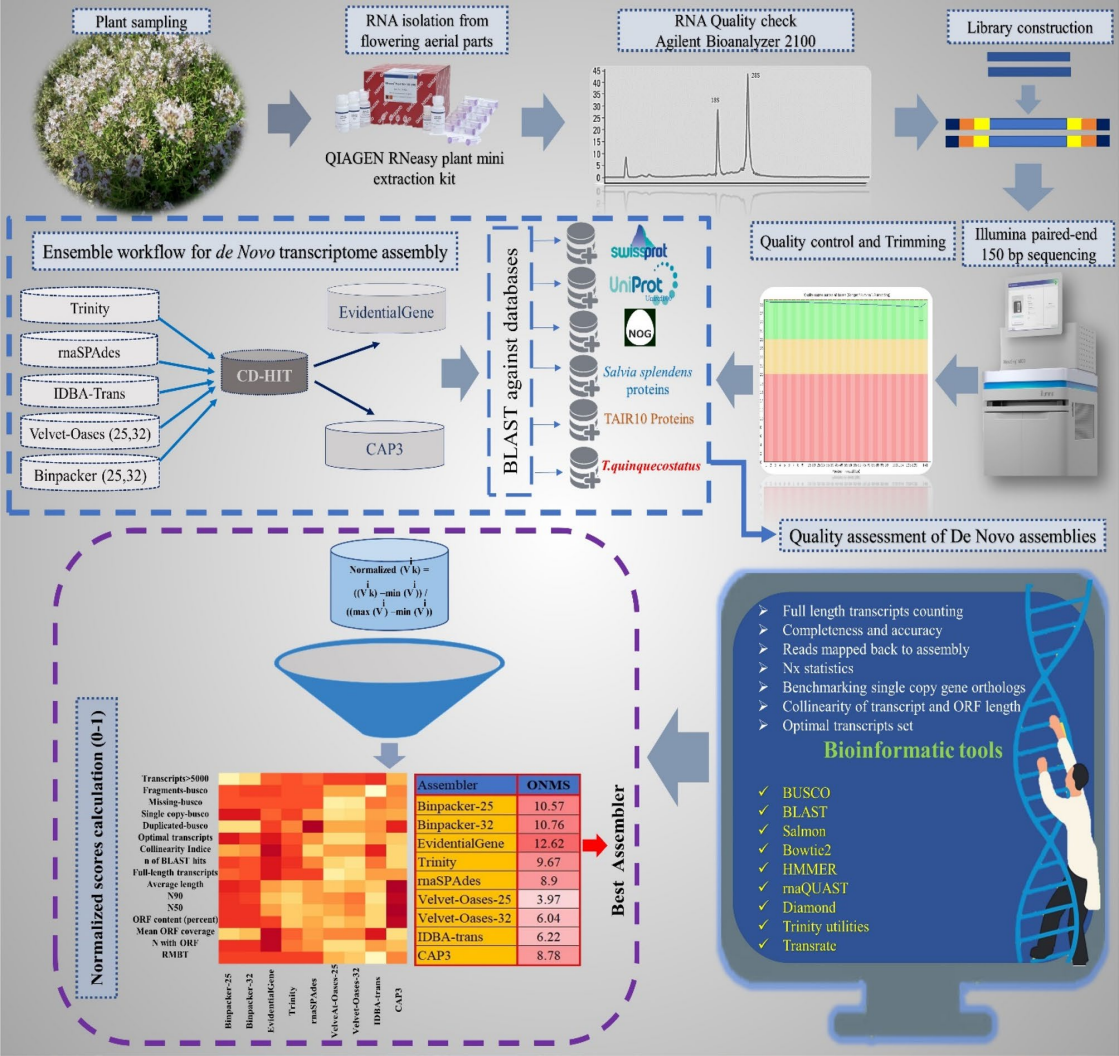
Developed Workflow for optimizing de novo transcriptome assembly and benchmarking the accuracy of different de novo transcriptome assemblers.

**Figure 2:**
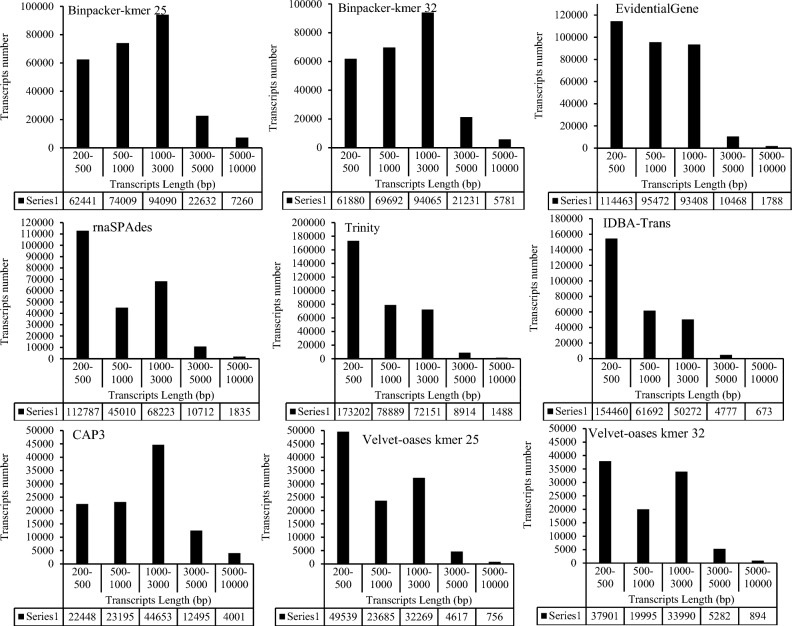
The transcripts length distribution as constructed by different assemblers.

**Figure 3:**
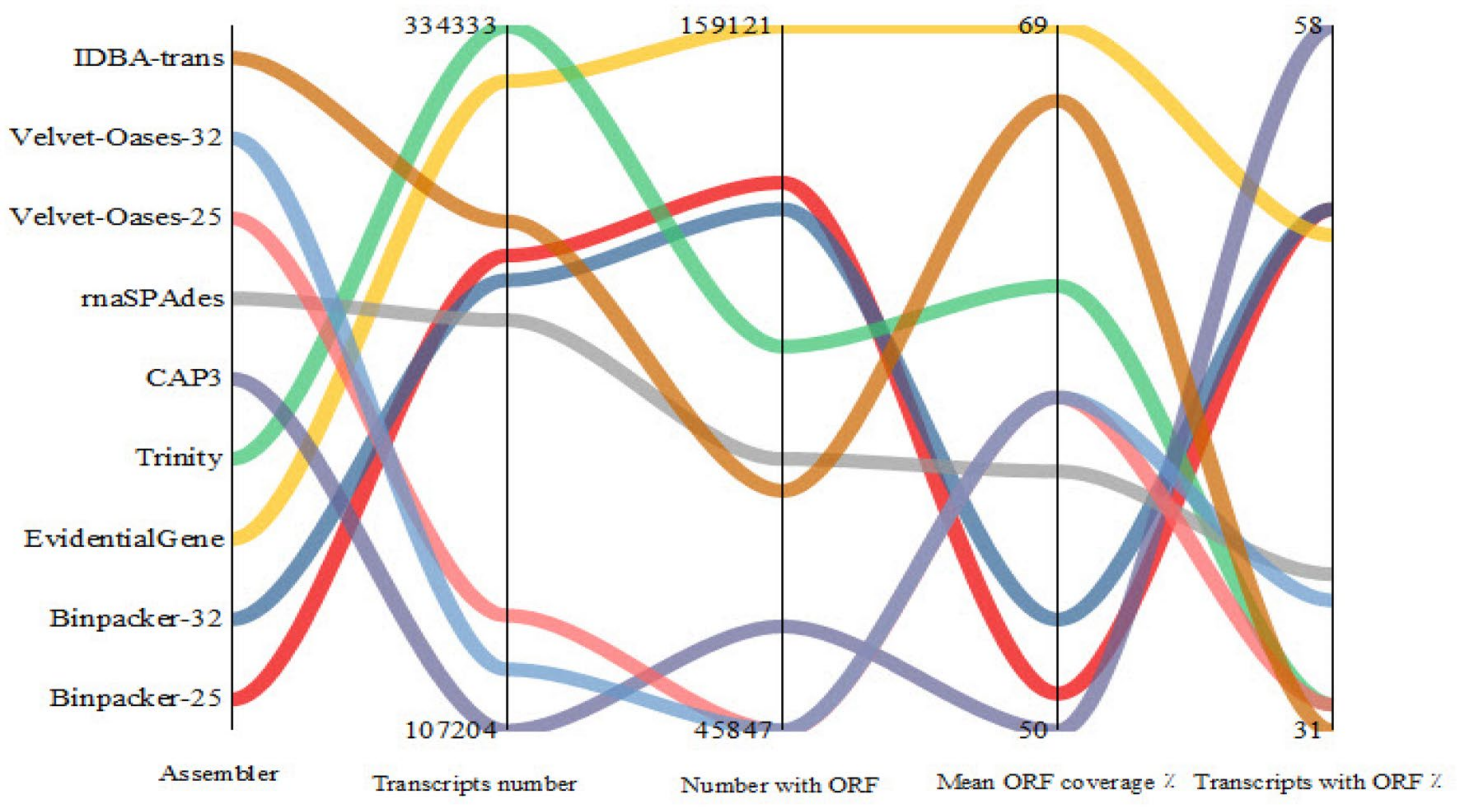
Comparison of different assemblers in terms of quantity of reconstructed transcripts, number of transcripts with a predicted ORF, mean percentage of transcripts coverage with predicted ORFs and percentage of transcripts that can potentially encode a protein.

After trimming, a total of 97,734,816 high-quality (Q>30) paired-end reads were obtained from the experiment, accounting for ~30 Gb nucleotide data. The trimming statistics and multi-FASTQC results are shown in Table S1 and Fig. S1, respectively. Following the ensemble workflow, the mRNA-seq data were subjected to nine de novo assembly series by utilizing seven well-known programs (Fig. 1). The size of the assemblies varied between 115 and 375 Mb (Table 1). The largest size of transcriptome assembly was related to BinPacker k-mer-25 (375 Mb), while the Velvet-Oases (111 Mb) assembler produced the smallest size (Table 1). Trinity (334,333) and EvidentialGene (316,786) could harvest the highest transcript numbers, whereas the assembly file of velvet-oases k-mer -32 had the lowest transcripts content (126,969) (Table 1). Velvet-Oases also generated the largest transcript (65,329 bp) (Table 1). The number of transcripts in Trinity’s output was about 2.6 times more than Velvet-Oases. According to Table 1, the highest number of potentially misassembled transcripts (i.e., over 5 kb and 10 kb) were found in the assembly results of BinPacker at both k-mer sizes. EvidentialGene and BinPaker k-mer-25 could recover the largest quantity of reconstructed transcripts over 500 bp (201,044, EvidentialGene) and 1 kb (124,376, BinPacker-25), respectively (Table 1). Analyzing the output of TransRate software disclosed that the highest number of optimal transcript sets (between 500 and 3500) is obtained through BinPacker k-mer-25 (198,276) and EvidentialGene (193,652). Velvet-Oases showed the lowest members for optimal transcript sets (Table 1). Nx statistics and average transcripts length were substantially different among transcriptome assemblers, as indicated in Table 1. The largest N50 values were designated for CAP3 (2561) and Velvet-Oases k-mer-32 (1924) assemblers, respectively. The results demonstrated that N50 enlarged parallel to the enhancement of k-mer size in Velvet-Oases. In addition, CAP3 (1678), BinPacker (at k-mer-25 (1441) and k-mer-32 (1402)), and EvidentialGene (986) were ranked respectively among the first three for the average length of transcripts. Interestingly, the results suggest that assemblers have a different operation for reconstruction of transcripts sets at different lengths (Fig. 2). Regarding Fig. 2, EvidentialGene gave a higher transcripts number in the length range of 500 bp and below 3000 bp compared to BinPacker or other assemblers. In fact, EvidentialGene was tagged in a beneficial way to harbor a reduced number of long over-assembled transcript sets within the ranges of 3000–5000 bp and 5000–10,000 bp, performing better than BinPacker. Except for BinPacker and CAP3, the other assemblers collected the majority of their transcripts in the 300–500 bp length range (Fig. 2). As illustrated by Fig. 3, 159,121 transcripts of EvidentialGene, accounting for 50% of whole transcripts, possessed at least one predicted open reading frame (ORF). The highest mean length coverage of transcripts with the identified ORFs was found in the EvidentialGene assembly. However, the highest percentage of ORF-containing transcripts (59% of total transcripts) and the lowest mean ORF coverage (50%) was obtained by CAP3. Velvet-Oases showed the lowest values for quantity and percentage of ORF-containing transcripts. Although IDBA-trans had a relatively high mean ORF coverage (67%), a minimum value (31%) was obtained for the percentage of transcripts with an ORF (Fig. 3). It should be noted that the BinPacker assembly containing a high number of transcripts with ORF (~ 130,000) did not yield a higher mean value for ORF coverage (53%) (Fig. 3).

**Table 1:** Statistics items and surveyed metrics for different de novo transcriptome assemblers for *T. daenensis.*

Assembly programs									
Metrics	BinPacker Kmer-25	BinPacker Kmer-32	EvidentialGene	Trinity	CAP3	rnaSPAdes	Velvet-Oases-25	Velvet-Oases-32	IDBA-trans
N of Transcripts	260,485	252,528	316,786	334,333	107,204	239,635	144,362	126,969	271,563
Over 500 bases	198,276	190,851	201,044	161,387	84,467	125,720	61,319	60,159	117,360
Over 1 kb	124,376	121,261	105,709	82,595	61,308	80,788	37,673	40,195	55,737
Over 5 kb	7667	5986	1843	1541	4168	1869	789	922	689
Over 10 kb	411	207	56	55	167	36	33	32	17
No of Optimal transcripts (500-3500bp)	198,276	172,471	193,652	155,168	72,787	117,924	58,096	56,314	114,211
N90	655	646	438	332	818	364	406	495	303
N70	1422	1402	881	717	1725	1054	1081	1272	590
N50	2265	2173	1441	1313	2561	1715	1764	1924	1091
N30	3405	3193	2135	2075	3650	2526	2621	2782	1779
N10	5677	5156	3631	3557	5806	4049	4723	4728	3116
Largest transcript	18,719	25,540	39,963	15,682	25,538	15,640	65,329	48,353	15,039
Mean length	1441	1402	986	816	1678	983	761	874	719
Assembled bases (Mb)	~ 375	~ 354	~ 312	~ 272	~ 180	~ 235	~ 115	~ 115	~ 195
GC content	44	44	45	43	44	44	44	44	44

### Percentage of reads mapped back to constructed transcriptome

**Figure 4:**
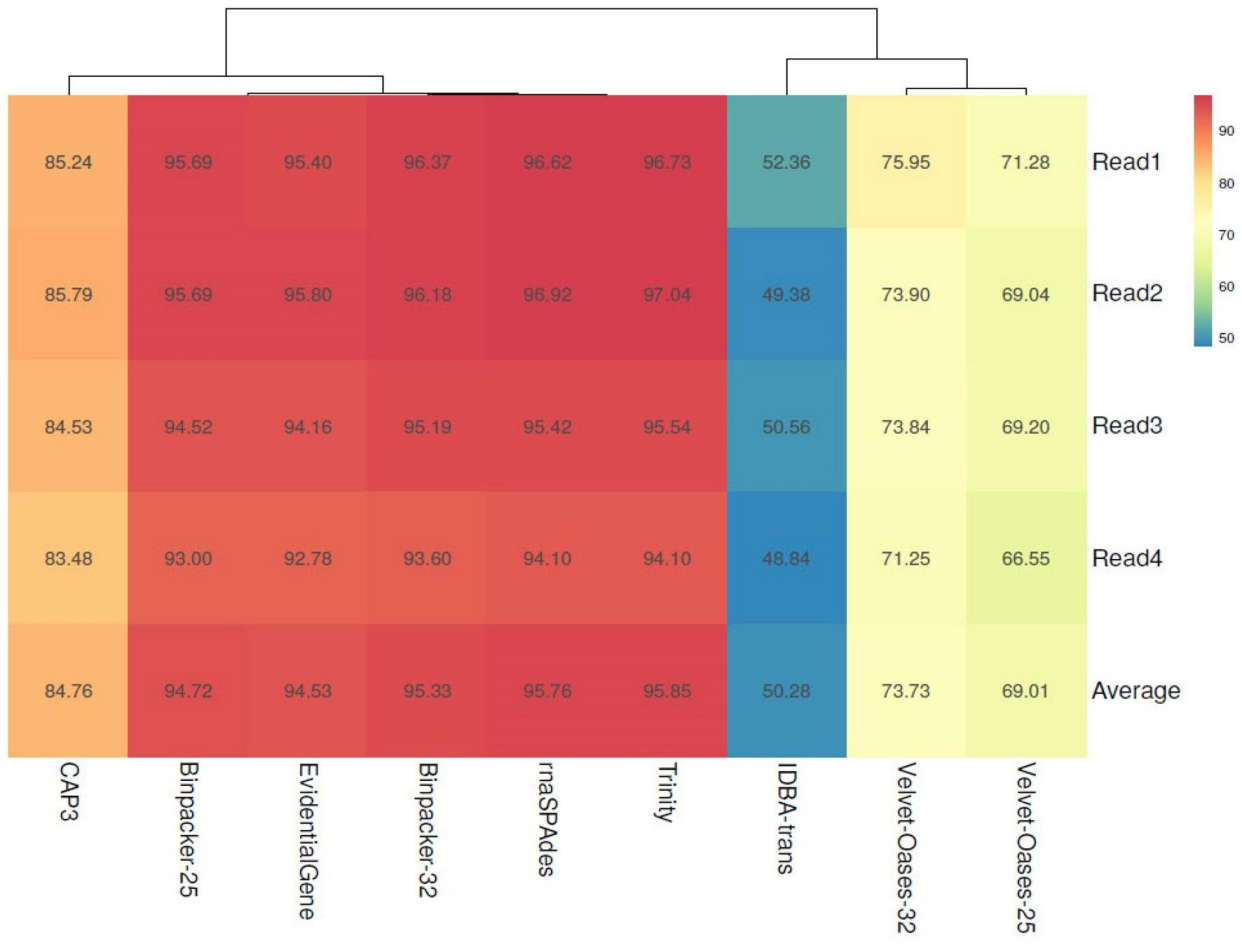
Value-labeled (percent) heatmap of reads mapped back to transcriptome assemblies (RMBT).

As depicted in Fig. 4, the assembly generated by Trinity exhibited the highest average reads mapping rate (95.85%). However, there was minor and trivial difference between Trinity and the other five assemblers (i.e., EvidentialGene (94.53%), rnaSPAdes (95.76%), and BinPacker (94.72% and 95.33%)) in term of mapping rate. IDBA-trans had the lowest mapping rate (50.28%) (Fig. 4). The percentage of reads mapped back to transcriptome assemblies for CAP3, Velvet-Oases k-mer-25, and Velvet-Oases k-mer-32 were recorded 84.76%, 73.73%, and 69.01%, respectively (Fig. 4). Based on these results, the application of a higher k-mer value (k-mer-32) for BinPacker and velvet oases could give a slightly higher mapping rate. Overall, there was no considerable difference between the mapping rate of samples (reads) (Fig. 4).

### Transcript’s completeness and length coverage

**Figure 5:**
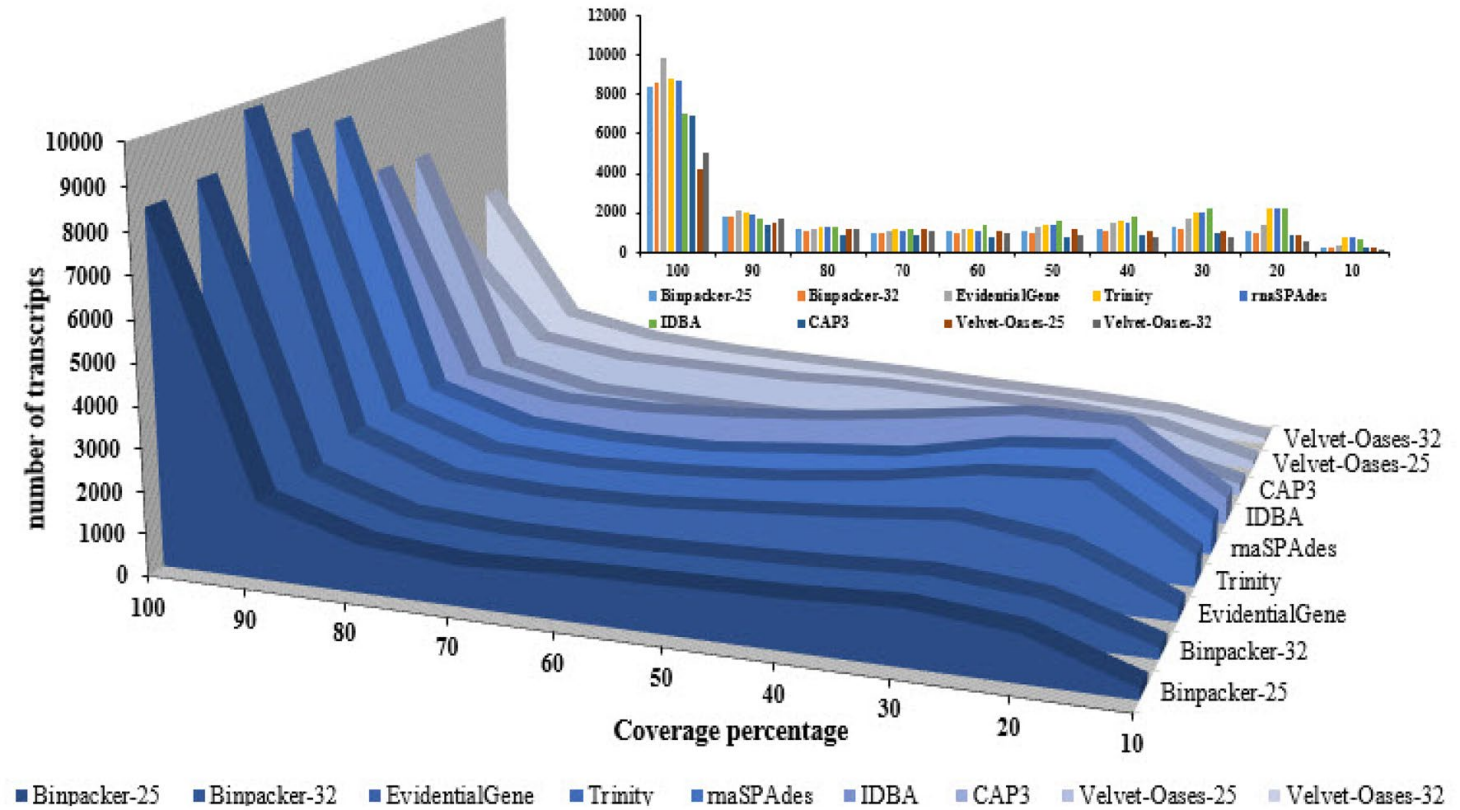
The number of protein coding transcripts rebuilt by assemblers that potentially support different levels of coverage.

The results revealed that EvidentialGene (9825), Trinity (8843), rnaSPAdes (8769), BinPacker-32 (8592), and BinPacker-25 (8408) possessed the highest number of full-length transcripts (90–100% coverage), while velvet-Oases assembly at both k-mer sizes had the lowest full-length transcripts (Fig. 5). There were also 2072 proteins that each matched a transcript of EvidentialGene by > 80% and < = 90% of their protein lengths (known as 90% coverage) (Fig. 5). Beyond the length coverages between 10 and 90%, Trinity could manifest roughly more transcripts than that of EvidentialGene (Fig. 5).

### Benchmarking universal single copy orthologues (BUSCO)

**Figure 6:**
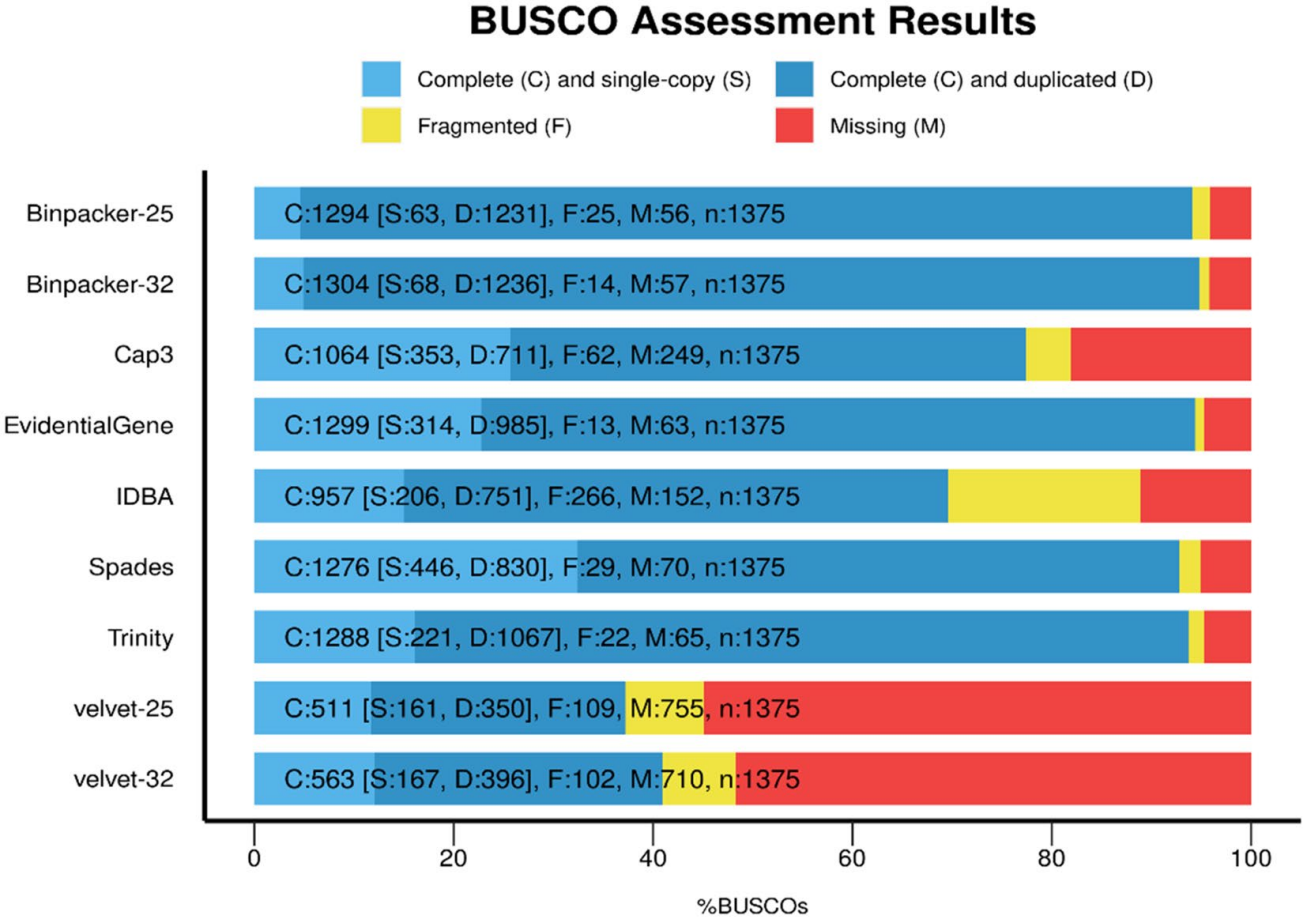
Comparing completeness of different assemblies based on benchmarking universal single-copy gene orthologs (BUSCO).

The outcomes of BUSCO analysis highlighted notable differences between transcriptome assemblers in terms of completeness. We were witnessed that the assembly outputs of BinPacker k-mer-32 (1304 [95%]), EvidentialGene (1299 [94.5%]), and BinPacker k-mer-25 (1294 [94%]) contained the maximum number of complete universal gene orthologs among assemblers (Fig. 6). Trinity (1288 [93.6%]), rnaSPAdes (1276 [92.8%]), CAP3 (1064 [78%]) and IDBA-trans (957 [70%]) were the next rich assemblers in term of complete gene orthologs (Fig. 6). Velvet-Oases at k-mer-25 (511 [41%]) and k-mer-32 (563 [37%]) represented the lowest content of complete universal gene orthologs (Fig. 6). Furthermore, rnaSPAdes (464 [32.4%]), CAP3 (353 [25.7%]) and EvidentialGene (314 [22.88%]) showed the highest number of complete and single copy orthologs (Fig. 6). EvidentialGene (13), BinPacker k-mer-32 (14) and Trinity (22) were also found to have the minimum number of fragmented gene orthologs. IDBA-trans (266 [19.3%]) exhibited the highest fragmented proportion (Fig. 6). The lowest content of missing universal gene orthologs were related to BinPacker (~ 0.040%) and EvidentialGene (0.045%). Taken together, it can be inferred that the greater k-mer-32 value could represent a more complete transcriptome assembly than k-mer-25.

### Collinearity of transcripts length and ORF size

**Figure 7:**
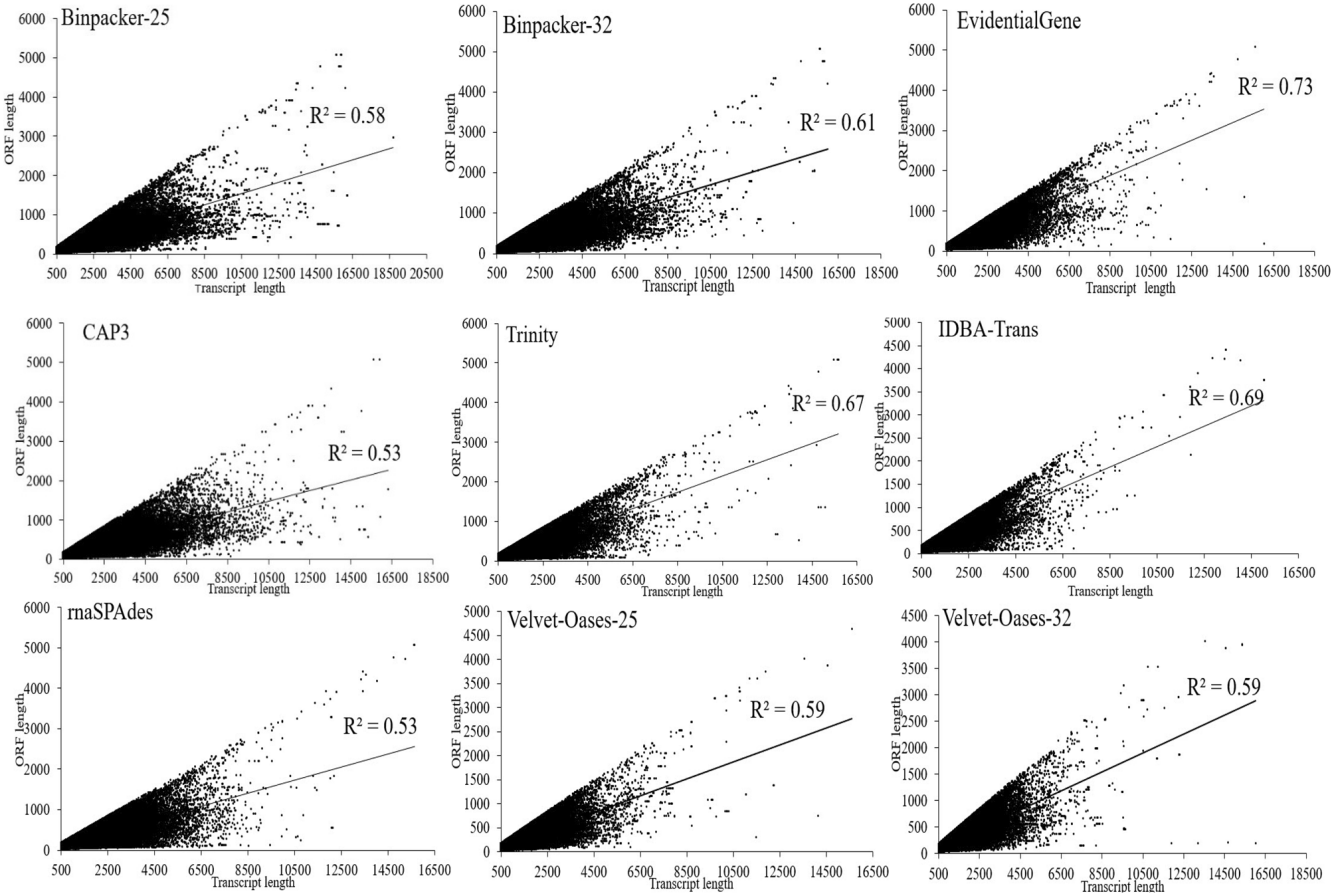
Scatter plots showing co-linearity of transcripts length and open reading frames (ORF) size in each assembler.

To evaluate the relationship between transcripts size and ORF lengths, linear models were fitted on these data. The results manifested that the scatter plot of EvidentialGene represents higher collinearity (R^2^ = 0.73) between transcripts length and ORF sizes (Fig. 7). Then, IDBA-trans (R^2^ = 0.69) and Trinity (R^2^ = 0.67) displayed high collinearity values (Fig. 7). BinPacker at k-mer-32 (R^2^ = 0.61) showed a slightly higher R-squared value than BinPacker k-mer-25 (R^2^ = 0.58), whereas there was no significant difference between R^2^ of Velvet-Oases results driven at k-mer 25 and 32 (R^2^ = 0.59). As shown in Fig. 7, CAP3 (R^2^ = 0.53) and rnaSPAdes (R^2^ = 0.53) had the weakest performance in establishing a linear relationship between transcripts and ORFs length (Fig. 7).

### Functional annotation

On average, uniref100 (163,038), *Thymus quinquecostatus* proteins (160,496), and eggNOG (151,994) databases showed a better function for the identification of assembled transcripts (Table 2). Moreover, One of the well-annotated genomes of the mint family belonging to *Salvia splendens* indicated a plausible performance in predicting the transcript function (Table 2). The predicted proteins of *S. splendens* had 149,044 BLAST hits on average. Referring to Table 2, EvidentialGene (213,502), Trinity (200,408), and BinPacker k-mer-25 (186,058) could generate the highest number of BLAST unique hits on average. The smallest number of BLAST unique hits were belonging to Velvet-Oases and CAP3. However, the largest proportion of identified transcripts (% of total transcripts) was found in CAP3 and BinPacker k-mer-25. Furthermore, the smallest proportion of the identified transcripts was related to Velvet-Oases. Similar to BinPacker, the outcomes indicated that applying the upper k-mer value (k-mer 32) in Velvet-Oases resulted in less identification of the transcript’s function. BLAST against Uniref100 database could uniquely assign a protein hit for a total of 235,730 (75%) and 225,383 (67%) transcripts related to EvidentialGene and Trinity, respectively. Interestingly, BLAST against *T. quinquecostatus* proteins as the closet species to *T.daenensis* within the same genus could yield close BLAST hit quantities compared with the Uniref100 database as one of the heaviest protein databases. The superiority of EvidentialGene was also proved based on the results of *T. quinquecostatus* proteins, as the assembler gained 6416 and 150,413 transcripts more than Trinity (as the most popular assembler) and Velvet-Oases (the weakest tool in the present study), respectively.

**Table 2:** Number of unique BLASTx hits obtained from searching transcriptome assemblies against five protein databases.

Assemblers	*Thymus quinquecostatus*	*Salvia splendens*	Tair10	SwissProt	EggNOG	Uniref100	Average blast hits	Identified transcripts based on Uniref100 (% of total transcripts)
BinPacker-25	198,651	188,593	175,901	161,779	190,720	200,707	186,058	0.77
BinPacker-32	191,890	180,616	167,623	153,489	181,711	191,497	177,804	0.75
EvidentialGene	231,828	215,269	196,107	180,998	221,084	235,730	213,502	0.74
Trinity	225,412	204,234	178,754	162,864	205,804	225,383	200,408	0.67
rnaSPAdes	160,661	150,134	137,925	131,163	163,516	177,224	153,437	0.74
Velvet-Oases-25	93,731	87,004	77,553	6,9194	83,542	89,589	83,435	0.62
Velvet-Oases-32	81,415	74,657	67,208	59,907	72,159	76,941	72,047	0.60
IDBA-trans	174,766	159,268	139,835	129,498	164,869	181,608	158,307	0.66
CAP3	86,117	81,629	76,777	72,109	84,542	88,668	81,640	0.82
Database average	160,496	149,044	135,298	124,555	151,994	163,038	147,404	0.71

### Evaluation of normalized metrics and multivariable analysis

**Figure 8:**
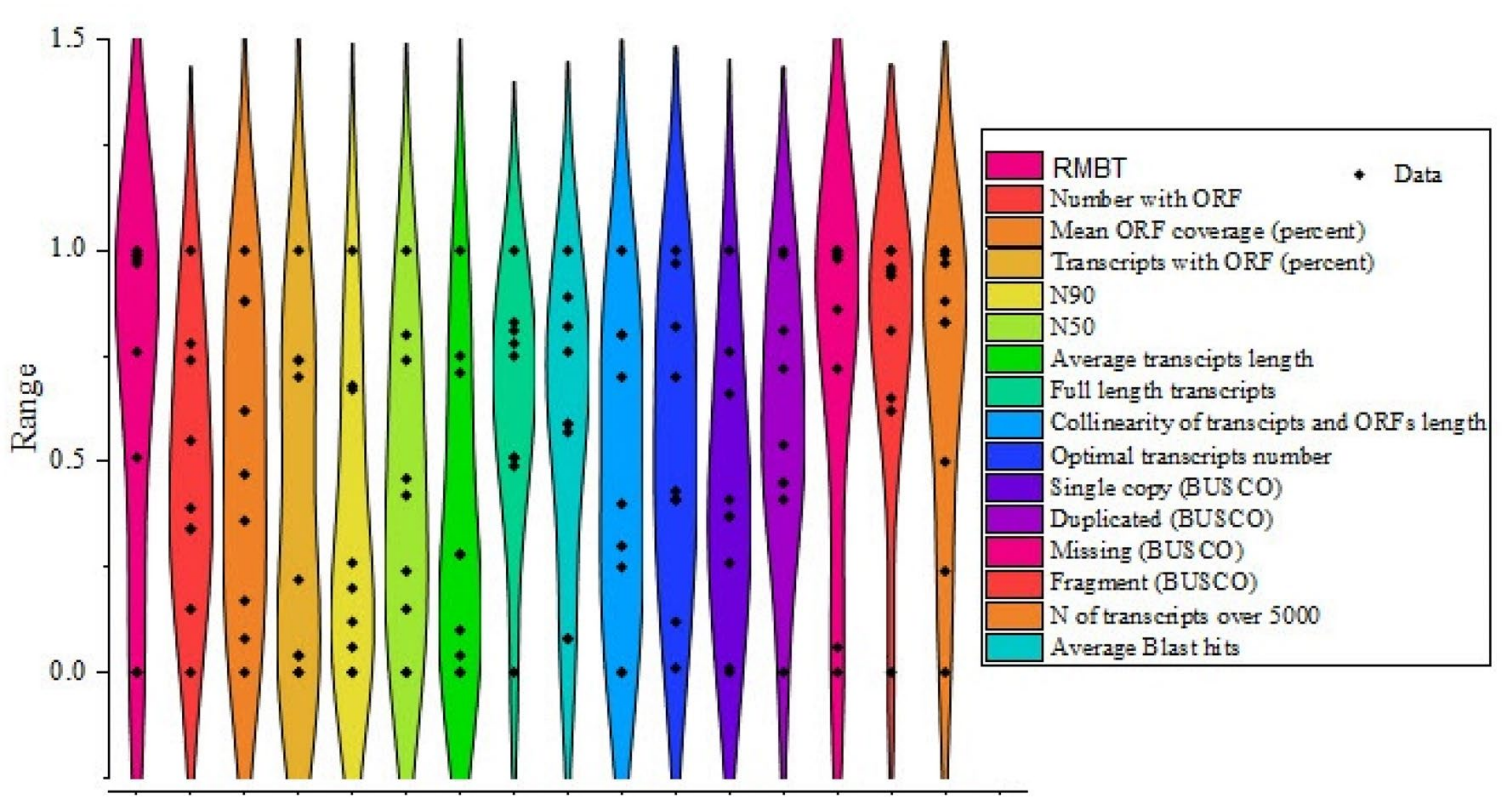
Violin plot showing the range of normalized scores of assemblers in the examined parameters.

**Figure 9:**
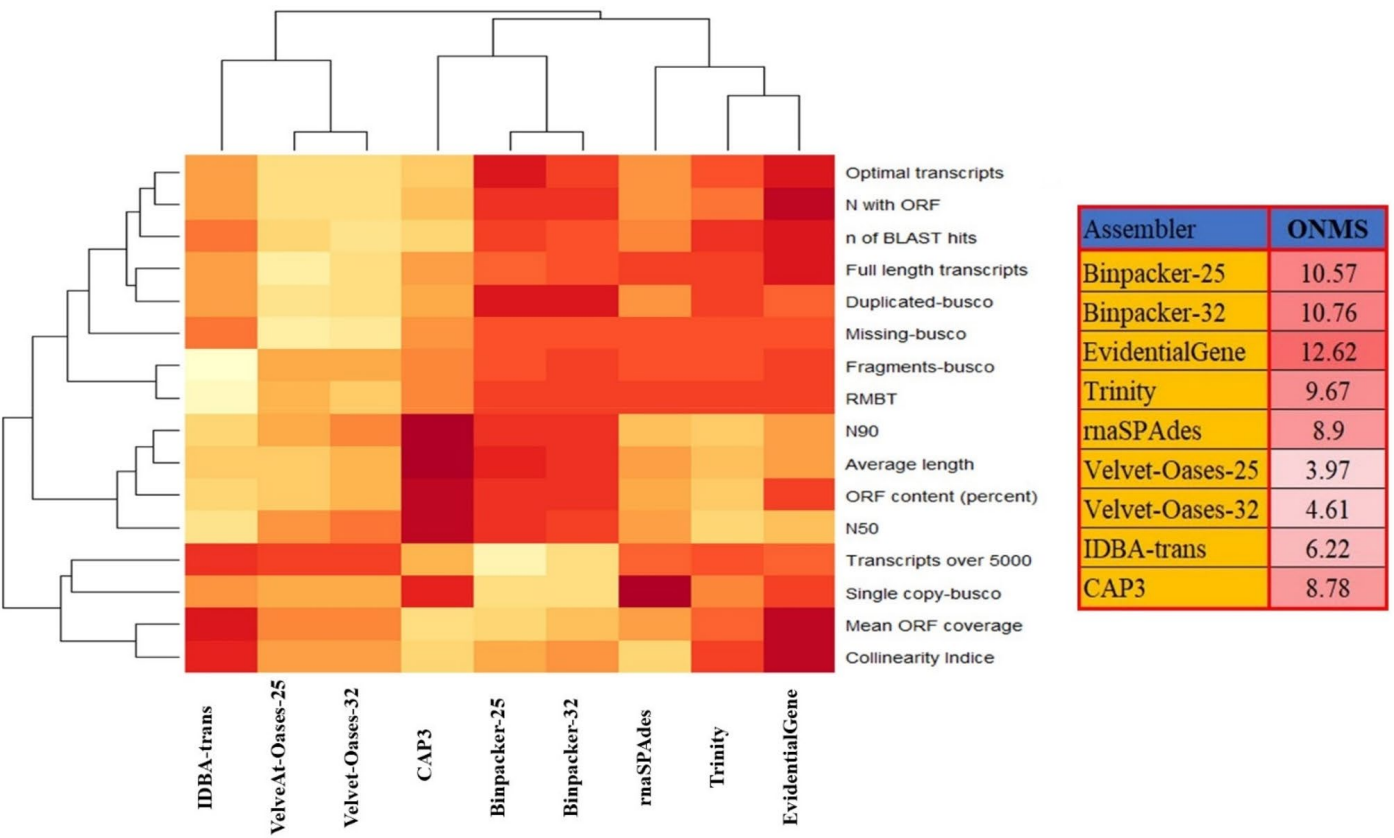
Heatmap plot indicating the overall normalized metric score (ONMS) and normalized scores (between 0 and 1) of each metrics obtained by each assembler.

Data normalization allowed us to compare assemblers and detect the best-performing tools. Zero stands for the weakest result, whereas number 1 denotes the best result. The higher values obtained by some quality-control characteristics such as missing or fragmented-BUSCO and transcripts greater than 5000 bp reversely received negative scores. Violin plot revealed that the values recorded for the evaluation metrics such as RMBT, average BLAST hits, missing-BUSCO, fragmented-BUSCO, and number of transcripts > 5000 bp were almost greater than 0.5 (Fig. 8). The value ranges of the other metrics were also between 0 and 1 (Fig. 8). Taking a quick look at the heatmap plot, EvidentialGene seems to have excelled in most evaluation metrics by acquiring the highest normalized scores (1) (Fig. 9). The overall normalized score (ONMS) (obtained by summing individual normalized scores) revealed that the EvidentialGene package (12.26 out of a possible 16) is the most effective tool for transcriptome assembly (Fig. 9). In the following, the moderate-performance assemblers BinPacker (10.57 and 10.76), Trinity (9.67), rnaSPAdes (8.9) and CAP3 (8.78) could achieve the next highest scores, respectively (Fig. 9). IDBA-trans (6.22) and Velvet-Oases (3.97 and 4.61) displayed the weakest performance (Fig. 9).

**Figure 10:**
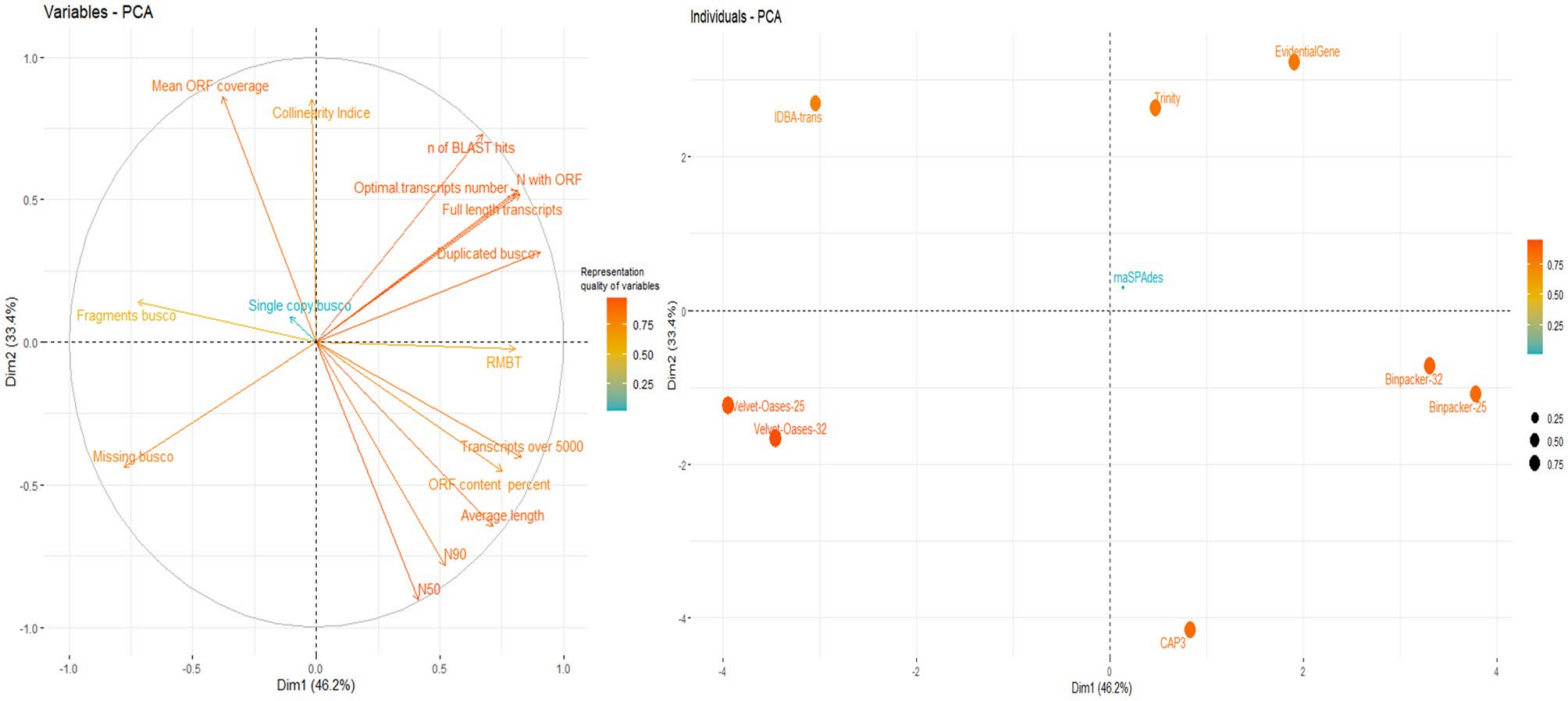
Principal component analysis (PCA) exhibiting the relationship between the examined metrics and assemblers.

A matrix composed of 144 data (16 variables × 9 observations) was used to conduct principal components analysis (PCA). The first two components explained ~ 80% of the total variance. The first dimension accounted for 46.2% of the total variance, while the second dimension accounted for 33.4% of the total variance (Fig. 10). CAP3, Velvet-Oases k-mer-25, IDBA-trans, BinPacker k-mer-25, and EvidentialGene could provide the largest contributions to dimensions 1 and 2 (Fig. 10). Indices such as N50, number of BLAST hits, number of transcripts with ORF, optimal transcripts and average length showed the highest quality of representation. Notably, rnaSPAdes showed the lowest contribution to dimensions. Principal component analysis (PCA) reaffirmed ONMS results by indicating that most of the metrics are oriented towards EvidentialGene (Fig. 10). BinPacker showed a strong correlation with RMBT and the number of transcripts greater than 5000 bp (Fig. 10). Furthermore, the biplot exhibited that the mean ORF coverage is in a close position with IDBA-trans assembler.

**Figure 11:**
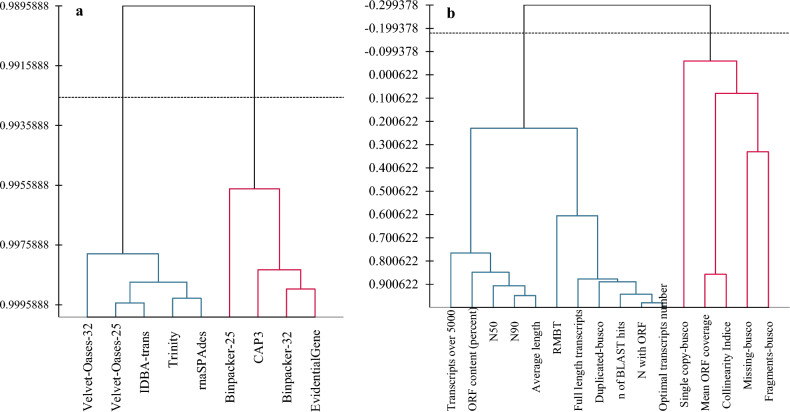
Agglomerative hierarchical clustering (AHC) based on similarity matrix classifying assemblers and measured metrics.

Based on the similarity matrix, agglomerative hierarchical clustering (AHC) categorized the assemblers into two main groups (Fig. 11a). Group I consisted of EvidentialGene, CAP3, and BinPacker. Group II hosted the other assemblers Trinity, IDBA, rnaSPAdes, and Velvet-Oases according to their common features (Fig. 11a). Meanwhile, AHC could classify the quality parameters into two main groups according to their discriminant functionality. Corresponding characteristics such as mean ORF coverage, collinearity indices, and completeness-related indices (BUSCO) were placed in the same group. This group was separated into three distinct sub-clusters (Fig. 11b). In the second group, the remaining indicators were well-divided into two main sub-clusters according to their differentiations (Fig. 11b). The first subcluster included N50, N90, average length, and ORF content (% of total transcripts) which were positively correlated with CAP3 and BinPacker. The second subcluster composed some metrics such as RMBT, optimal transcript sets, number of full-length transcripts, BLAST hits, transcripts with ORF, and duplicate gene orthologs. The aforementioned metrics were highly correlated with the EvidentialGene package.

### Gene ontology and trans-decoder analysis on the best performance assembler

In addition to BLAST annotations against six protein databases, we performed a gene ontology and trans-decoder analysis on EvidentialGene as the best assembler to provide a detailed annotation and re-verification its obtained results. According to Fig. S2, transcripts were categorized in three groups. In the first category (biological process, 55.82%), cellular process, metabolic process, organic substances metabolic process, primary metabolic process and single organism processes were the most prominent Go terms. In the second category (molecular function, 18.17%) binding and catalytic activity got the highest number of GO terms. Terms such as cell, cell parts, intracellular and organelle parts were in top rank positions of cellular components category (31%). Moreover, the analysis of coding potential of EvidentialGene output through trans-decoder software indicated that a total of 225,088 transcripts had coding frames (Fig. S3). The number of transcripts possessing complete ORFs (86,424), 5-prime partial ORF (60,023), internal ORF (53,043), and 3-prime partial ORF (25,598) is shown in Fig. S3.

## Discussion

As a matter of fact, genome assembly and transcriptome analysis are complementary and these procedures do not take place of each other^29^. The genome assembly encompasses the structural data, information on chromosomes, SNPs, and haplotypes^6–9^. However, it is still so expensive, labor demanding, time-consuming, and needs high computational resources^6–9^. For non-model organisms such as *T. daenensis* which has no reference genome, the transcriptomic data can provide a quickly-obtained information on its overall genes and reconstructed transcripts. Compared with genome assembly, transcriptomics can assist in identifying different kinds of splicing isoforms from a unique gene, quantification of gene expressions, and finding new genes not reported in preliminary genomes^8,9,29^. In fact, the investigation of an intermediate stage between genes and proteins, that is, mRNA transcripts, makes it possible to bridge the gap between the genetic codes and the functional molecules by connecting the genome to genes function^6,7^. That is why despite having a reference genome accessible, it is still advisable to perform a de novo transcriptome analysis in order to identify any transcripts that may have been overlooked during the genome assembly process^8,26^.

Without subsequent pre-processing procedures, such as clustering the concatenation results, the final transcript sets may contain several redundant sequences, primary transcripts, incomplete isoforms, chimeric artifacts, and gene fusions^7^. In general, the ensemble approach primarily attempts to enhance the information-rich sequences while avoiding an inflated transcriptome assembly by removing redundancy and enriching coding regions^29^. Within the mRNA-seq read pool, where an uneven coverage depth exists, applying higher k-mer values would theoretically consolidate the possibility of constructing a complete assembly and contiguous transcripts, especially in the case of abundantly expressed isoforms^34^. Exerting k-mer-32 setting in Velvet-Oases (developed for genome assembly) could produce lengthy transcripts with a higher value for N50 and average length, as confirmed by other studies^26,34^. However, this did not take place in BinPacker k-mer-32. Usually, applying a larger k-mer in Velvet-Oases ends with long transcripts. Because this program is intuitively sensitive to abundant transcripts and usually builds longer contigs from highly presented sequences^34^. Schulz et al.^17^ stated that the use of short k-mer sizes may increase the risk of introducing over-assembled transcripts, but it may meaningfully improve the collection of rare transcripts. Similarly, the CAP3 assembler was able to construct the longest transcripts with high average length and Nx values due to its innate merging nature^29^. Technically, CAP3 creates longer consensual sequences based on similar isoforms that exceed predetermined thresholds^29^. The underlying implications support that the underestimated or exaggerated N50 values can be due to the following reasons: (1) short and fragmented transcripts (below 500 bp); (2) very long misassembled transcripts; (3) chimerism and (4) highly expressed genes^34^. Misassembled transcripts often include insertions or deletions, frameshifts, unclear UTR regions, and messed coding frames^35^. Chimerism, another frequent phenomenon in transcriptome assemblies, can typically originate from either artificial (assembler error) or natural source^8^. Gene fusion, trans-splicing, and polymerase read-through mechanisms for adjacent genes are the most commonly known implications of chimerism^8^. In contrast to plant species, large proteins (~ 10,000 aa) have previously been found in animal muscle^35^. The largest reasonable protein length in plant clades is expected to be about 1500 aa (maximum transcript size = 4500–5000 bp)^35^. Li et al.^13^ demonstrated that the majority of long transcripts (> 5 kb) found in different transcriptome assemblies were aligned with several accessions and indicated less query coverage. The authors also reported that for over 80% of transcripts < 500 bp, there were no counterparts in the protein databases ^13^. From a biological perspective, some reports corroborate that most of the full-length transcripts in plant species range from 500 to 3500 bp^36–39^. In the current research, EvidentialGene ended up with a low level of redundancy, small number of potential mis-assemblies (over 5 kb), higher optimal transcript sets (0.5–3.5 kb), and full-length transcripts. The assembly output of EvidentialGene could also represent a higher ORF number and mean ORF coverage. By contrast, BinPacker produced more numbers of probably over-assembled transcripts (> 5000 bp) than other assemblers. The above-stated computational precedence of EvidentialGene comes from the point that this software works with amino-acid sequences and concordantly checks CDS quality, UTRs length, gaps, and the presence of start/stop codons^35^. The software primarily tries to keep the best CDS and UTR in perfect duplicates and retains the longest CDSs in perfect fragments as well^35^.

Inferences support that the basic parameters such as N90, N50, mean length, or the total number of transcripts may not be sufficient for assembly assessments. These findings call for proactive tracking of these indicators and research into other determinants. Indeed, these parameters do not estimate the completeness of the assembly in terms of gene content^40^. Comparative analysis of different assemblies based on universal single-copy gene orthologs is another useful strategy that can be helpfully utilized. Single-copy genes are also frequently used in phylogenetic studies because they are repeatedly restored to a single state under selection pressure due to their resistance to duplication events^41^. Out of 1375 single copy gene orthologs, 1294, 1304, 1299, and 1288 complete orthologs were respectively present in the outputs of BinPacker-25, BinPacker-32, EvidentialGene, and Trinity. It may be concluded that these assemblers are able to provide reliable completeness for transcriptome assemblies. However, some assemblers examined here (IDBA-trans and velvet-oases), had a high proportion of fragmented or missing lineage-specific orthologs. The high ratio of fragmented or missing gene orthologs can be implied to the computing weaknesses of these programs in defining a true boundary for the isolation of false negative transcripts^26^. The findings of our study well-matched with those results of Sheikh-Assadi et al.^26^ and Stander et al.^39^, who demonstrated that Velvet-Oases and IDBA-trans had the worst performance in reconstructing a complete transcriptome compared with other assemblers. Pavlovikj^42^ examined the efficiency of three assemblers (velvet-oases, SOAPdenovo, and trinity) with multi k-mer options on de novo transcriptome assembly of wheat. The author concluded that velvet-oases performed as the best assembler and found that multi k-mer runs in all three assemblers gives better overall assembly result. These findings suggest suitability of each assembler relies on species^26,43^. Another critical factor that should be carefully monitored is the mapping rate back to the transcriptome, which significantly affects downstream analyses, such as the abundance estimations and gene enrichment analysis^44^. We found that two assemblers (IDBA-trans and Velvet-Oases) were unable to warrant a credible mapping rate for downstream paths. Recent studies have confirmed that IDBA-trans and Velvet-Oases cannot produce reliable mapping rates for the RNA-seq data of other plant species^26,45^. Aside from these two assemblers, the other tools could yield justifiable results. Of these tools, CAP3 and EvidentialGene displayed reduced levels of redundancy because their reads were more uniquely mapped to one transcript. It is worth noting that both EvidentialGene and CAP3 displayed a high value for the collinearity of transcript size and ORF length. It is evident that as the transcript lengthens, a higher ORF size is expected. The collinearity results of assemblers were well-consistent with their given mean ORF coverage values. The ORF coverage shows how an open reading frame covers the length of a transcript. By accumulating good results from each assembler, EvidentialGene could eventually win this competition by getting the highest normalized overall score. This superiority was also verified by generating the highest number of unique BLAST hits. Based on the BLAST results, annotating transcripts using *T. quinquecostatus* proteins showed close and similar quantities to Uniref100 database. This result suggests that using genome-derived proteins of *T. quinquecostatus* can be a time-effective, plausible and alternative procedure to annotate *T.daenensis* transcripts or other species of *thymus* rather than using heavy databases such as Uniref100 or NR NCBI. As the second step of the ensemble approach, CD-HIT clusters transcripts based on their similarity and removes the redundant sequences prior to the third step. Then, EvidentialGene pipelines translate the transcripts codons to amino acid sequences and focus on choosing the best transcripts (based on amino acid quality, stop/start codons, and UTRs) among similar gene isoforms within a pre-clustered data. The software also removes duplicates and the very similar transcript isoforms. Indeed, the resultant transcripts have a certain level of non-redundancy and each gene has its unique isoforms. Therefore, one transcript isoform (uni-transcripts) corresponds to one unique protein hit.

Overall, BinPacker or Trinity could achieve a relatively high ONMS values. Hence, impatient people using these single assemblers would receive partly satisfactory results. Our findings emphasize that it is best not to use a single assembler for all species, no matter how common it is. As this study showed, BinPacker can easily corner the widely-used assembler, Trinity, in creating the *T.daenensis* transcriptome.

## Material and methods

### Sampling, RNA isolation, and mRNA-sequencing

Four samples from flowering aerial parts of vegetatively propagated *T. daenensis* plants were sourced from Medicinal Plant Collection of Department of Horticultural Science, Karaj (35°77′ N, 50°92′ E). Then the samples were fast-frozen using liquid nitrogen. Dr. Vahideh Nazeri, the botanist, identified plant samples and accessions were deposited at Herbarium of the Department of Horticultural Science under voucher specimen number 16543-16546. Then, samples were transferred to a −80 °C freezer until RNA extraction. Total RNA was isolated using Qiagen RNeasy plant mini extraction kit (QIAGEN, Hilden, Germany) according to manufacturer instructions. The integrity of 18S and 28S ribosomal bands were checked on 1% agarose gel. The purity and quantity of RNA samples were analyzed with NanoDrop 100 (Themofisher Scientific, USA). The RNA integrity was also re-checked to pass the quality control step (Agilent 2100 Bioanalyzer, USA). Briefly, samples with RNA integrity numbers greater than eight were submitted for poly(A) capture and cDNA library construction using the TruSeq standard mRNA preparation kit (Illumina company, CA, USA). Finally, 150-cycle pairwise sequencing was carried out on the Illumina sequencing machine.

### Quality control and data trimming

The status of the sequencing reads was initially screened using FastQC tool v0.11.9. Bases quality, adaptor contamination, and overrepresented sequences were checked^46^. Before transcriptome assembly, raw data were preprocessed and filtered for low-quality reads by executing Trimmomatic v0.39^47^ program by setting ILLUMINACLIP:adaptors.fa:2:30:10:2:True, HEADCROP:10, LEADING:30, TRAILING:30, and MINLEN:50 parameters.

### Workflow of de novo transcriptome assembly

The entire workflow was performed on Ubuntu Linux (v20.04 LTS) supercomputer with a 22-threaded processor and about 400 GB of RAM. Seven publicly available assembly programs were employed to carry out a comparative benchmarking on these tools’ efficiency. Trinity v.2.15^17^ and rnaSPAdes v.3.15.5^20^ were driven with default k-mer parameter. Binpacker v1.0^27^ and Velvet-oases^18^ were run with two k-mer (i.e., k-mer 25 and 32) options. IDBA-trans^19^ was executed through a range of k-mers from 20 to 60 with an increment step of 10. The transcripts-60.fa file was used as the final output for this assembly. All of these seven assembly FASTA files (except the output of EvidentialGene and CAP3) were merged together to shape the ensemble approach. According to ensemble workflow, the merged transcript outputs of these seven assemblies were pre-processed through CD-HIT v4.8.1^48^. CD-HIT clusters and removes the exact transcript duplicates based on a pre-determined threshold (-c 0.99) before finalizing the ensemble workflow (Fig. 1). Since CAP3^29^ is not a multi-threaded program, the un-clustered data causes running to fail and get out of memory. On the other hand, tr2aacds pipeline of EvidentialGene package needs a pre-clustered data. Therefore, CAP3 as a consensus-based merging tool was subsequently driven on CD-HIT clustered data of combined assemblies with similarity threshold of 95%. In addition, the perl scripts trformat.pl, tr2aacds.pl and evgmrna2tsa.pl were executed consecutively on CD-HIT clustered data using the EvidentialGene v2018may07^35^ package utilities to acquire "main" and "alternative" transcription sets.

### Reads mapping rate

To determine the reads mapping rate, all paired-end trimmed reads were mapped back to each transcriptome assembly using Bowtie2^49^ aligner and by setting –very-sensitive mode.

### Assessment of transcripts length coverage

Constructed transcripts of each assembler’s output were aligned against the reviewed protein database SwissProt by calling the “diamond blastx”^50^ command and regarding these parameters: -max target seqs 1 and -evalue 1e-20. Then, the blastx outputs were applied to count the transcripts with variable length coverage using the perl script “analyze_blastPlus_topHit_coverage.pl” provided by Trinity utilities (https://github.com/trinityrnaseq/trinityrnaseq/wiki/Counting-Full-Length-Trinity-Transcripts).

### BUSCO analysis for transcriptome completeness

HMMER and NCBI’s tBLASTn utility were used through BUSCO v 5.4.4^40^ pipeline to find and annotate single-copy gene orthologs of assembly results by searching them against the embryophyta_odb10-lineage-specific dataset. The identified orthologs were finally visualized and classified as single-copy and complete, duplicated and complete, fragmented, or missing.

### TransRate and rnaQUAST: analysis and evaluation of basic metrics

To accomplish benchmarking of transcriptome assemblies on the basis of the fundamental statistics, rnaQUAST v 1.0.3^51^ and TransRate v2.2.2^52^ were employed. The rnaQUAST software listed the average transcript length, number of transcripts, and number of transcripts over 0.5, 1, and 10 kb. TransRate, as a reference-free quality assessment method, delivered basic metrics such as the number of assembled bases, Nx statistics, transcripts number with ORF, mean ORF coverage of transcripts (%), and other indices. The file generated by this software listing transcripts length and the predicted ORF length was used to create the scatter graphs.

### Functional annotation of assemblers

To assess annotatability, potential functions of *T. daenensis* transcripts, and also to count the number of BLAST unique hits, transcripts of each assembly output were used as queries to search against the reference protein databases. In this respect, BLASTX with an e-value of 1e-5 and one target sequence was done. The protein databases used for this purpose were Tair10 (*Arabidopsis thaliana*), *Thymus quinquecostatus, Salvia splendens*, Swiss-Prot, eggNOG, and Uniref100. Each of these nine assembly files was blasted against the databases mentioned above and a fully 54 BLAST run was conducted. Furthermore, Gene ontology classification of the best performance assembler (i.e., EvidentialGene) was conducted through online webserver (AgriGo: http://systemsbiology.cau.edu.cn/agriGOv2/). Transdecoder V5.7 analysis (https://github.com/TransDecoder/TransDecoder) was also conducted on EvidentialGene output to dissect the coding potentials of the transcripts.

### Final decision-making: calculation of normalized scores

The efficacy of de novo assemblers was evaluated by calculating normalized scores of 16 selected metrics (X) for each assembler. The normalization was carried out as follows: (X^i^_k_–Min (X^i^)) / (Max (X^i^)–Min (X^i^)). Where i represents the assembler, K represents the estimated metric, and X_i_^k^ denotes the actual value of the metric i determined for each assembler (K). Max or Min of X^i^ also represents the highest/lowest actual values of that the i metric of the given assembler. To achieve an ultimate perspective of each assembler performance, an overall normalized metric score (ONMS) was calculated by summing all of the normalized values for each assembler^53^.

### Ethics approval

All the procedures related to collection and multiplication of plant materials (sourced from Medicinal Plant Collection of Department of Horticultural Science, Karaj (35°77′ N, 50°92′ E)) were followed in accordance with Iranian department of environment (DOE) guidelines and permissions for collection of bio-samples were obtained from this authority. The study was approved by ethics committee of Horticultural Department of University of Tehran.

## Conclusion

This study advanced a redundancy-reducing de novo assembly approach for *T. daenensis* by integrating the features of nine single and combined de novo assembly programs. We have selected 16 important criteria for discrimination and determining effectuality of these assemblers. Using AHC analysis, the 16 selected criteria were correctly differentiated into two sub-groups, showing similar discriminatory behavior. Taken together, it is recommended that these criteria should be used to calibrate and select an optimized transcriptome assembly. The current work demonstrated that the EvidentialGene package could deliver the most satisfactory and concise results by acquiring the highest scores for the majority of evaluated indices. Interestingly, BinPacker gained the upper hand over Trinity and outperformed the other programs in most quality parameters studied. Nonetheless, CAP3, used as a consensual merger program, unexpectedly did not produce reliable results because it yielded a low mapping rate and produced very lengthy and possibly misassembled transcripts.

## Supplementary Information

Supplementary Information.

## Data Availability

The datasets generated and analyzed in the current project (PRJNA950567) is deposited in NCBI’s SRA repository and data are released publicly with the following accession numbers: (read 1: SRR24037549, read 2: SRR24039021, read 3: SRR24039245, read 4: SRR24040063).
