# Supplementary material for: Optimizing an efficient ensemble approach for high-quality de novo transcriptome assembly of Thymus daenensis
Source: Sci Rep. 2023 Jul 31;13:12415. doi: 10.1038/s41598-023-39620-6 (PMC10390528; doi:10.1038/s41598-023-39620-6)
Supplement: Supplementary file 1 — Supplementary Information. [file 41598_2023_39620_MOESM1_ESM.docx]

**Table S1**. Trimming results of FASTQ file obtained by Trimmomatic software.

| Samples | Total reads | Total paired reads | Forward unpaired reads (removed) | Reverse unpaired reads  (removed) |
| --- | --- | --- | --- | --- |
| Sample 1 | 25,590,414 | 25,590,361 | 17 | 36 |
| Sample 2 | 24,336,138 | 24,336,067 | 52 | 19 |
| Sample 3 | 23,569,775 | 23,569,741 | 28 | 6 |
| Sample 4 | 24,238,704 | 24,238,647 | 42 | 15 |


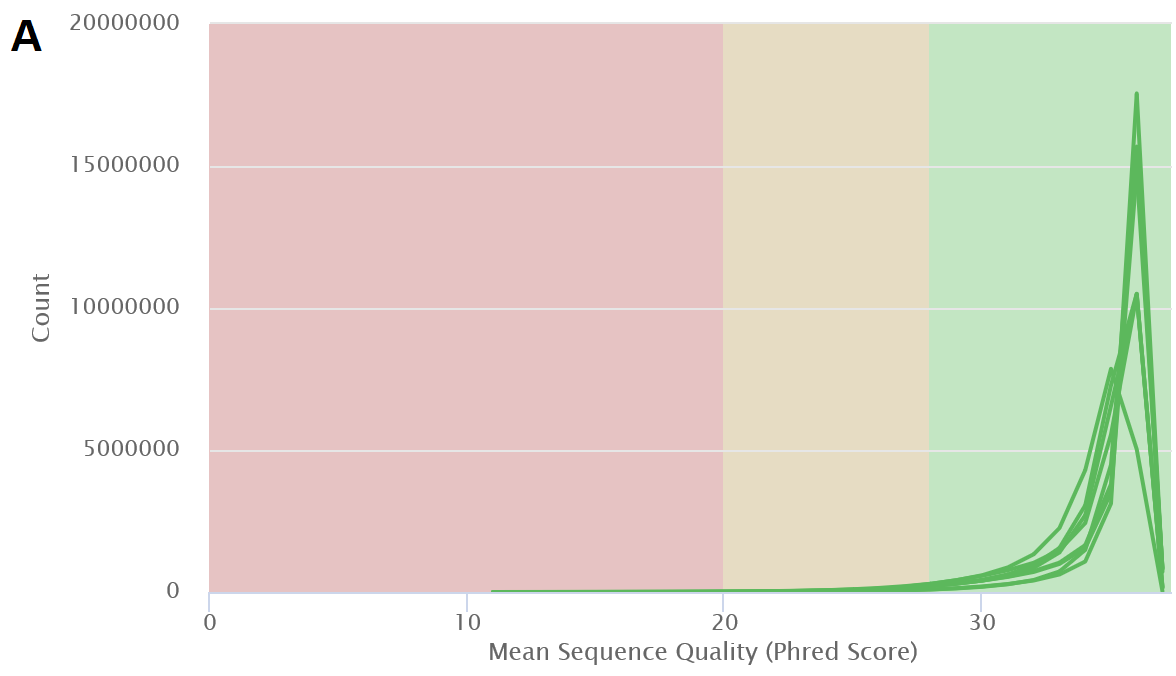

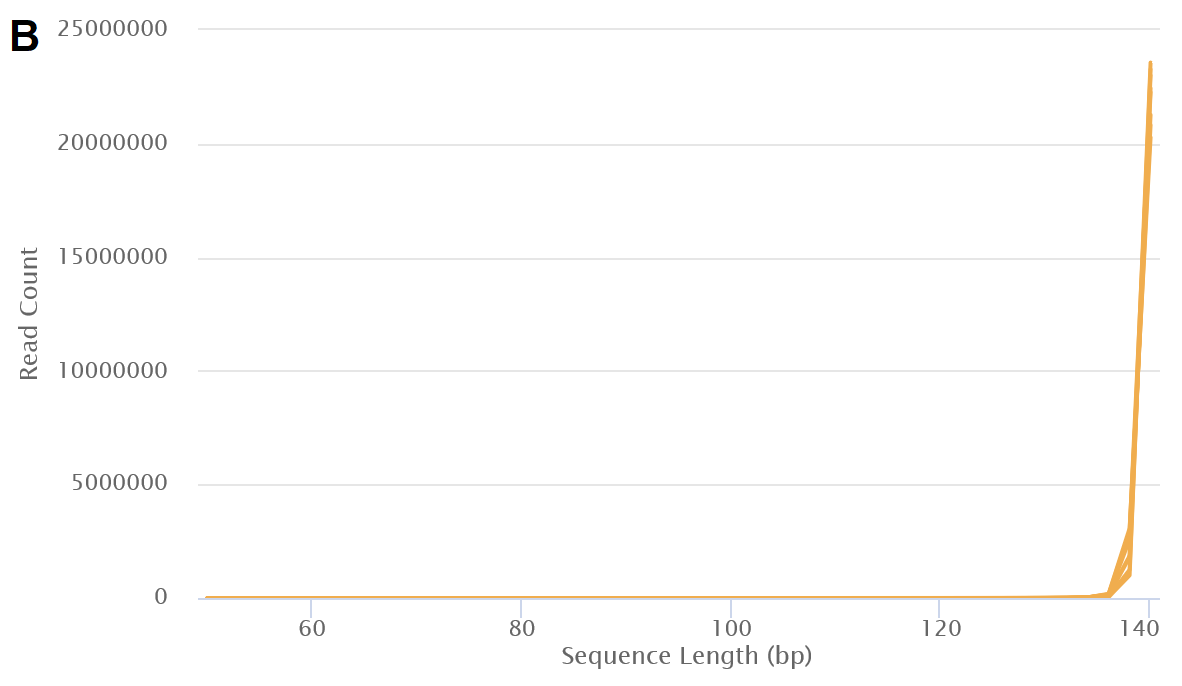


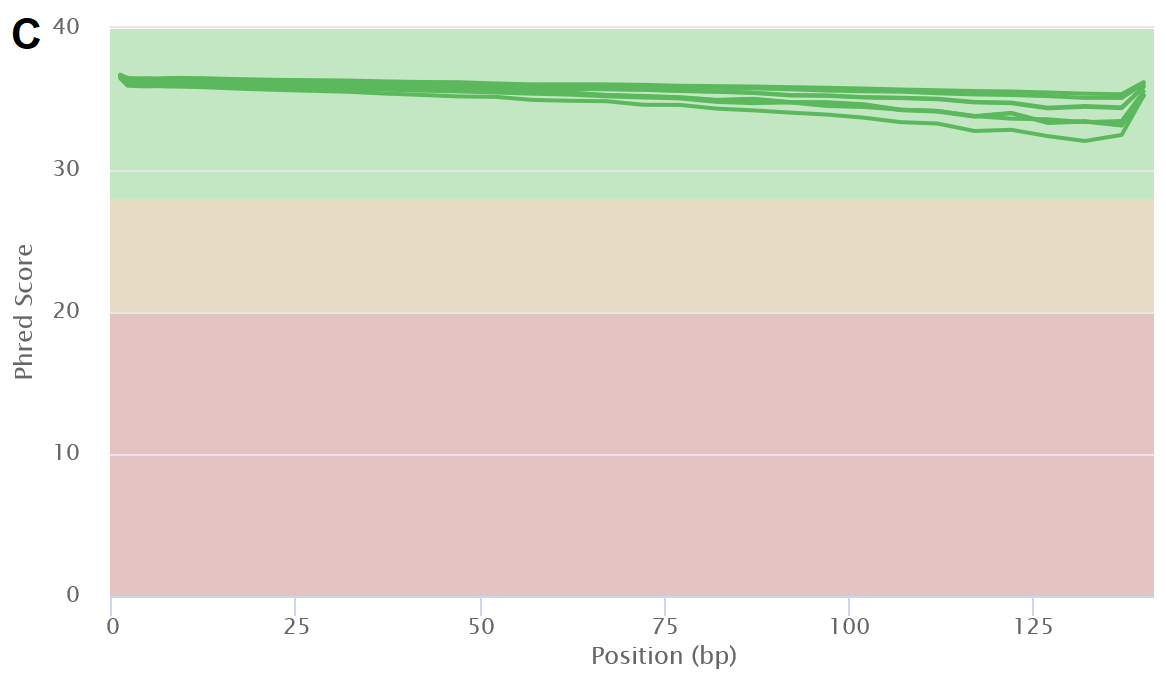


**Fig S1.** Multi-FASTQ quality check results for the trimmed RNA-Seq data related to reverse and forward files of experimental samples. (**A**) Per sequence quality scores. (**B**). Sequence length Distribution (**C**). Mean quality scores.

**Fig S2.** Gene ontology classification of EvidentialGene results as the best performance transcriptome assembler.

**Fig S3.** Gene ontology classification of EvidentialGene results as the best performance transcriptome assembler.
